# Enzyme-driven triplex structure-based DNA logic circuits

**DOI:** 10.1186/s12951-025-03853-6

**Published:** 2025-12-24

**Authors:** Xiao Liu, Jing Zhang, Xuehao Zhang, Zhuo Chen, Shize Zhang, Xinyu Wang, Jiaying Liu, Zhiheng Wang, Liyi Xie, Zhihao Ming, Longjie Li, Kaixiong Tao, Xianjin Xiao, Xiaoyu Zhou, Yu Xie

**Affiliations:** 1 Hunan Cancer Hospital, The Affiliated Cancer Hospital of Xiangya School of Medicine, Central South University, Changsha, 410000 China; 2 Institute of Reproductive Health, Tongji Medical College, Huazhong University of Science and Technology, Wuhan, 430030 China; 3 Department of Biomedical Sciences and Tung Biomedical Sciences Centre, Department of Precision Diagnostic and Therapeutic Technology (CityUHKMSRI), Key Laboratory of Biochip Technology and Biotech and Health Centre (CityUSRI), City University of Hong Kong, Hong Kong, 999077 China; 4 Department of Gastrointestinal Surgery, Union Hospital, Tongji Medical College, Huazhong University of Science and Technology, Wuhan, 430030 China; 5 School of Pharmacy, Tongji Medical College, Huazhong University of Science and Technology, Wuhan, 430030 China; 6 Department of Breast Surgery, Second Hospital of Jilin University, Changchun, 130000 China; 7 School of Life Science and Technology, Wuhan Polytechnic University, Wuhan, 430023 China

**Keywords:** DNA computing, DNA logic circuits, DNA nanotechnology

## Abstract

DNA logic circuits have emerged as crucial media for biological computing due to their molecular-level parallelism and high biocompatibility. In the two mainstream systems currently prevalent, the strand-displacement system generally suffers from issues such as high strand complexity, signal leakage, and difficulties in cascading. In comparison, the enzyme-driven system has offered a further improvement in high rates and low leakage. However, it continues to encounter challenges pertaining to signal leakage and signal attenuation, which limit the development of intricate computational systems.This study introduces enzyme-powered triplex DNA logic circuits that simplify strand design through input-gate concatenation forming triplex structures, utilizing Bst 3.0 polymerase for efficient operation. System validation includes single-gate analysis, multi-level cascades (secondary/tertiary), complex logic circuits, and large-scale square root operations. Single-gate circuits achieve < 2-min half-completion times with minimal leakage. The two-level cascaded circuits exhibit minimal leakage and the longest half-completion time of less than 8 min, while 10-gate square root circuits maintain < 25-min operation with 24.3% reduced strand complexity and 25% faster reaction rates versus existing systems. The low-leakage architecture enables efficient DNA computing for biosensing, data storage, and synthetic biology applications, while its modular design supports scalable biological computing networks.

**Graphical Abstract:**

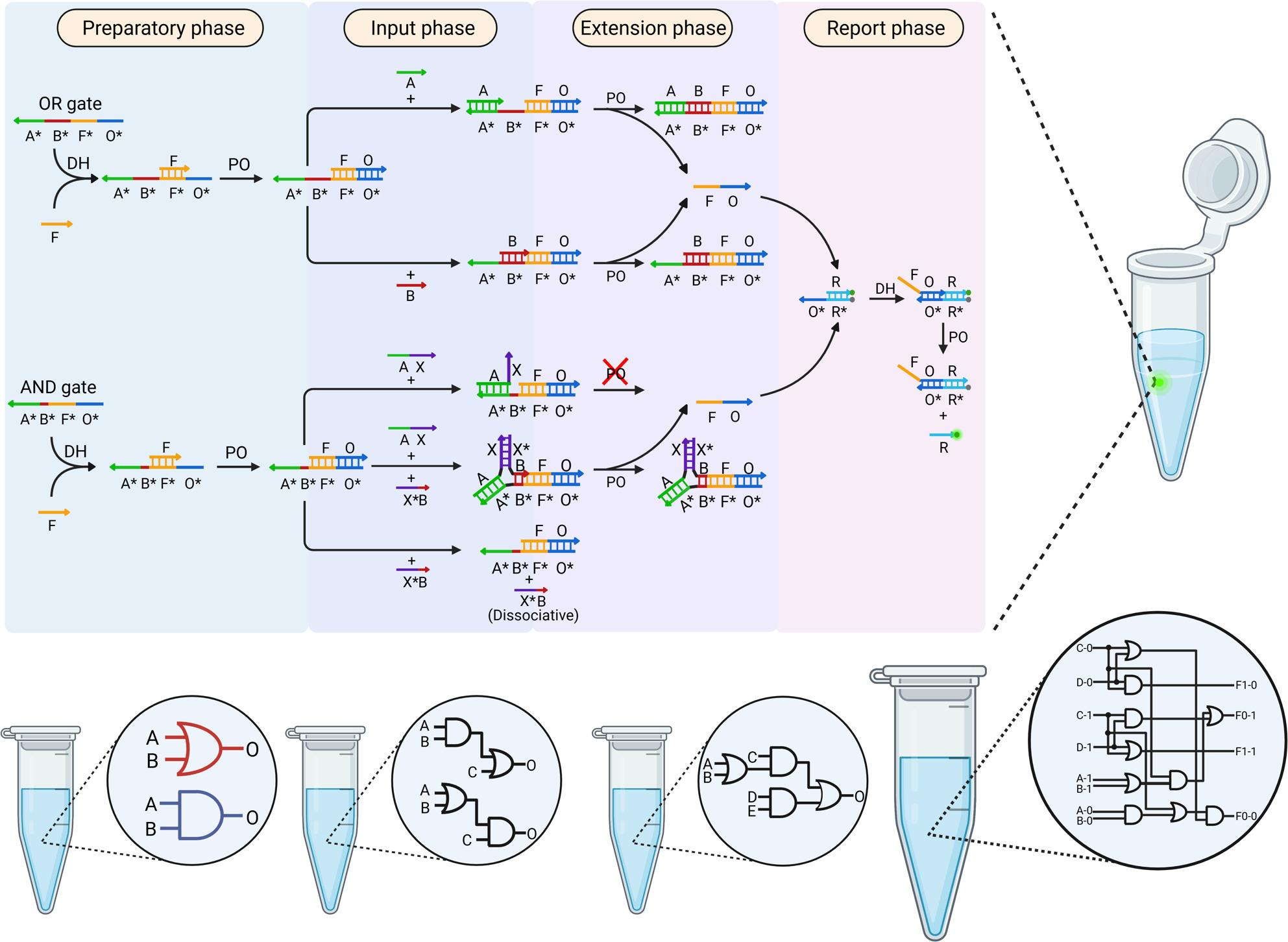

**Supplementary Information:**

The online version contains supplementary material available at 10.1186/s12951-025-03853-6.

## Introduction

Based on molecular-level information processing and continuous computation within biological systems, organisms exhibit a complex and fascinating diversity [1, 2]. Compared to traditional silicon-based materials, DNA stands out as an efficient and stable biological storage and computing medium due to its unique base-pairing properties, molecular-level parallelism, high biocompatibility, and responsiveness to environmental changes [3–5]. Leveraging these superior attributes of DNA, fundamental Boolean logic gates can be constructed to assemble DNA logic circuits for information processing and computation, addressing complex challenges effectively [6–8].

The field of DNA logic circuits has consistently been driven by the pursuit of systems capable of higher performance and the realization of more complex computational frameworks, with these advancements closely tied to the strand complexity inherent in the constructed systems [5, 9, 10]. Currently, DNA logic circuits can be primarily categorized into two major systems: strand displacement-driven and enzyme-driven. Each system has its unique characteristics, providing diverse options for the practical application of DNA logic circuits [4, 11].

Strand displacement-driven DNA logic circuits can construct basic logic gates and some simple cascading circuits [12–14]. However, due to their high strand complexity, they generally suffer from signal crosstalk and leakage when building large-scale logic circuits. For instance, the seesaw gate developed by Qian et al. (2011) exhibited severe leakage issues caused by its complex architecture [15]. Although the system constructed by Fan et al. (2020) significantly reduced strand complexity, noticeable leakage was still present in their result [16].

Enzyme-driven DNA logic circuits exhibit excellent computational performance with high rates and low leakage [17–19], making them the most cutting-edge system in current research [20–22]. It is important to note that special enzymatic reactions not only enhance performance but also reduce strand complexity in design, significantly improving the programmability and cascading potential of DNA logic circuits. However, issues such as signal leakage and signal loss remain prevalent. For instance, the system constructed by Su et al. (2019) can build large-scale logic circuits with high reaction speeds [23], yet signal leakage still occurs in large circuits. On the other hand, Song et al. (2019) developed logic gates composed solely of single strands, effectively addressing slow reaction rates and high strand complexity [24, 25]. Nevertheless, the substantial inherent signal loss in their AND gates poses challenges in constructing large-scale cascaded circuit systems [26–29]. Consequently, there is currently a lack of a DNA logic circuit system that combines low strand complexity with high performance.

Here, we introduce a novel enzyme-driven DNA logic circuit, the triplex structure-based DNA logic circuits, which combines low strand complexity with high performance. Compared to existing enzyme-driven systems, we further reduce the strand complexity in our design to construct a superior circuit system. By leveraging the simple concatenation of input strands to form triplex structures, we enable efficient logic operations while significantly reducing strand complexity. The system maintains high reaction rates and minimal leakage, as confirmed by individual testing of each logic gate to validate operational accuracy and performance. On this basis, we constructed secondary cascades (OR-AND, AND-OR, AND-AND, OR-OR), a tertiary AND cascade, and relatively complex cascaded logic circuits to demonstrate our programmability and cascading capabilities. Finally, we assembled a large-scale square root circuit with practical computational significance, comprehensively validating our triplex structure-based DNA logic circuits. The high-speed, low-leakage performance of cascaded circuits validates our design philosophy focused on minimizing strand complexity.

## Materials and methods

### Materials

Oligonucleotides used were purchased from Sangon Biotech (Shanghai). Oligonucleotides were checked by Sangon Biotech via capillary electrophoresis for purity and by electrospray ionization mass spectrometry for identity. All labeled strands (FAM, HEX, ROX, Cy5, BHQ-1, BHQ-2, and BHQ-3 labeled) were HPLC purified by Sangon Biotech. Oligonucleotides were ordered in a dry format and suspended in Tris − EDTA buffer. Concentrations were determined by measuring the absorbance at 260 nm using a NanoDrop 2000 spectrophotometer (Thermo Fisher Scientific). Bst3.0 polymerase and Isothermo Buffer(Mg^2+^ free) [20 mM Tris − HCl, 10 mM KCl, 10 mM (NH_4_)_2_SO_4_ and 0.1% Triton X-100, pH 8.8 at 25 °C] were purchased from HaiGene Biotech Co., Ltd. Exonuclease III was purchased from New England Biolabs.

### DNA sequence design

The sequence design principle of the DNA strands used in this work is illustrated in Supplementary Information Fig. S1.1, and their specific sequences are shown in Supplementary Information Section S7. The CG % are set to be around 40% to ensure appropriate melting temperatures (except for the poly A at the 3′ends). DNA strands used in the same reaction do not contain obvious secondary structures and undesired crosstalk at 50 °C. The sequence design was verified by NUPACK [30] to confirm their binding energy and specificity.

### Enzyme reaction

To run calculating process, 4 units of Bst 3.0 DNA polymerase (HaiGene Biotech Co., Ltd.), 1 mM of each type of deoxynucleotide (HaiGene Biotech Co., Ltd.), and 4 mM MgSO_4_ (HaiGene Biotech Co., Ltd.) were added. Subsequently, the PCR tube was inserted into a real-time qPCR machine (Hangzhou Bioer Technology Co., Ltd. or Hangzhou Lifereal Biotechnology Co., Ltd.) and incubated at 50 °C for 10 min to facilitate the synthesis of the output strands. Ultimately, the introduction of input strands marked the commencement of circuit operation.

### Fluorescence acquisition and data normalization

The output signal values were recorded by a real-time qPCR machine at 50 °C with the fluorescence intensity measured. Four channels were used: FAM (excitation 494 nm, emission 518 nm)-BHQ1(quenching range 480–580 nm), HEX (excitation 535 nm, emission 556 nm)-BHQ1 (quenching range 480–580 nm), ROX (excitation 585 nm, emission 605 nm)-BHQ2 (quenching range 550–640 nm), and Cy5 (excitation 643 nm, emission 667 nm)-BHQ3 (quenching range 620–730 nm).

Each fluorescence curve, representing an output of logic “1”, was normalized using its minimum value as 0 and its maximum value (average of last three data points) as 1. The normalization of logic “1” curves was executed individually to ascertain the half-completion time for each curve as our circuits lacked signal amplification processes and exhibited varying maximum values, even with identical reporter complexes. This differed from prior enzyme-free catalytic DNA circuits, wherein the maximum values of all logic “1” curves remained nearly constant with the same reporter complex. From a digital standpoint, this discrepancy did not hinder the functional performance of our circuits because in our experiments, the maximum values of all logic “1” curves were vastly higher than those of corresponding logic “0” curves. For each logic “0” curve, given the minimal fluorescence increase observed during the experiment, we selected the logic “1” curve from the same channel of the corresponding circuit and utilized its maximum value as the reference value of 1 for the logic “0” curve. In scenarios where multiple logic “1” curves were available, we opted for the one with the lowest maximum value to prevent artificial suppression of the logic “0” curve, thus ensuring a true reflection of the leakage.

## Results

### Design of single gates in triplex structure-based DNA logic circuits

**Fig. 1:**
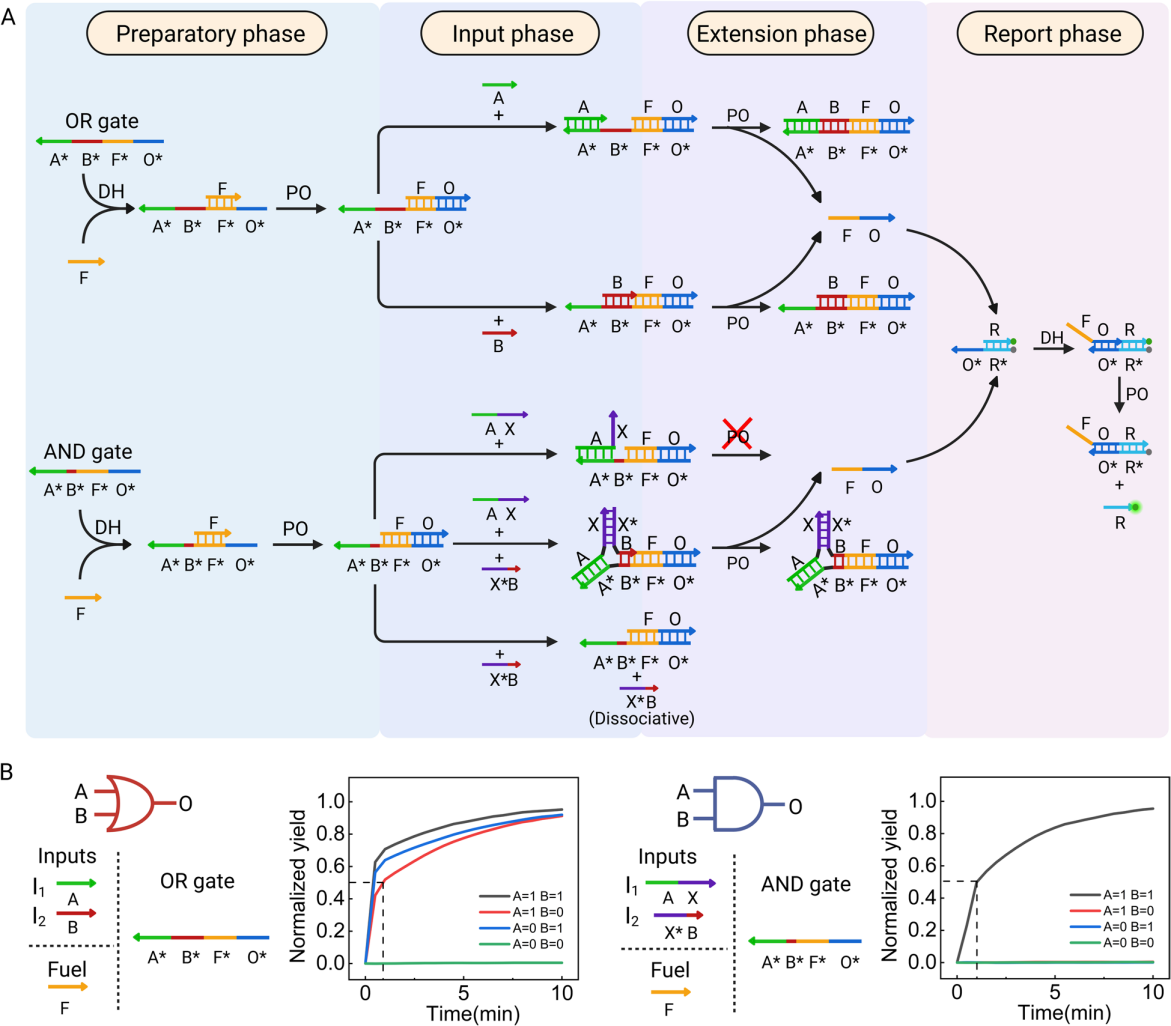
Design and performance of the OR and AND logic gates. **A** Schematic diagram of the reaction principle for OR and AND gates computing processes.The entire reaction consists of four phases: preparation, input, extension, and reporting. Elaborate the specific mechanisms of DNA hybridization (DH) and polymerization (PO) reactions occurring on a single gate. All reactions in this paper are performed at 50 °C. **B** Composition design of OR and AND gates and experimental results. Here and below, the truth tables for all logical operations can be found in the Supplementary Information Figs. S5.1 and S5.2. All data shown are from at least three independent experiments

The foundation for constructing DNA logic circuits lies in the assembly of AND and OR gates, as various logical operations and computational systems within the circuit can be built through the combination and connectivity of these gates. In our system, the OR gate consists solely of a single gate strand (A*B*F*O*) and a fuel strand (F). Compared to the OR gate developed by Song et al. (2019), our design integrates two gate strands into one, thereby reducing the strand complexity. Besides, the AND gate is composed of a single gate strand (A*B*F*O*) and a fuel strand (F). For the inputs, unlike other systems where inputs are typically composed of a single domain, our AND system's inputs are formed by two domains. Specifically, input I_1_ (AX) is comprised of an A domain and an X domain, while input I_2_ (X*B) consists of an X domain and a B domain. Notably, the X* domain is capable of complementary binding with the X domain, and the B domain is exceptionally short, encompassing a mere 6 nucleotides (Fig. 1B).

The operation principles of OR and AND gates are illustrated in Fig. 1A, consisting of four sequential phases: preparation, input, extension, and reporting. During the preparation phase, the fuel strand F is added into the reaction system containing the gate strand. The F strand binds to the F* site on the gate strand through DNA complementary base pairing. Subsequently, Bst 3.0 DNA polymerase initiates DNA polymerization and extension to form the FO strand, resulting in the intermediate complex structure (A*B*F*O*:FO). Bst 3.0 DNA polymerase has 5' to 3' DNA polymerase activity and strong strand displacement activity toward DNA or RNA templates but lacks 5' to 3' and 3' to 5' exonuclease activity.

The next stage is the input phase. For the OR gate: when input I_1_ is present, the A domain on I_1_ binds to the A* domain on the gate strand, providing a 3' end for the next extension step; when input I_2_ is present, it functions similarly to input I_1_; When both inputs I_1_ and I_2_ are present, their binding order to the gate strand gives rise to two distinct reaction pathways. Although both pathways ultimately generate effective output, the prior binding of I_2_ leads to the formation of an intermediate complex (BFO-O*F*B*A*), which can subsequently react with I_1_ to produce additional output strands. Consequently, this competitive binding does not suppress the output signal but may, in fact, enhance it (The specific reaction pathway is shown in Supplementary Information Fig. S1.3). For the AND gate: when only input I_1_ is present, the A domain of I_1_ binds to the A* domain on the gate strand, while the X domain fails to bind to the gate strand, resulting in no 3' end available for DNA polymerase extension, stopping at the input phase and failing to enter the extension phase, thus no output is generated. When only input I_2_ is present, although the B domain on input I_2_ theoretically can bind to the B* domain on the gate strand, due to the extremely short length of the B domain on input I_2_, it cannot support stable binding between input I_2_ and the gate strand under the reaction temperature of 50 °C. This results in free input I_2_ and intermediate complexes still present in the system, with no 3' end available for DNA polymerase extension. The process halts at the input stage, failing to generate any output (Further details about the length of the B domain are provided in Supplementary Information Fig. S2.1). However, when both inputs I_1_ and I_2_ are present, the A domain of I_1_ binds complementarily to the A* domain on the gate strand. Simultaneously, input I_2_ can rely on its own X* domain to bind with the X domain of input I_1_, enabling input I_2_ to utilize this interaction to stabilize the binding of its B domain with the B* domain on the gate strand. This results in the formation of a triplex structure through concatenation, providing a 3' end for DNA polymerase to proceed with the next extension step.

The process proceeds to the extension phase: under the action of Bst 3.0 DNA polymerase, each structure extends along its respective 3' end. During this extension process, FO strand is dissociated from the complex, resulting in free single-stranded FO. The O domains within the free FO single strands are utilized as the output for individual gates.

Next, the system enters the reporting phase: the displaced FO strand reacts with the reporter complex (composed of two strands containing a quencher and a fluorophore, respectively). The O domain of the FO strand binds complementarily to the O* domain of the reporter complex, and upon extension by DNA polymerase, the strand containing the quencher is displaced. This allows the fluorophore to generate a fluorescent signal, enabling the monitoring and visualization of the output for each gate. The step-by-step and detailed basic logic gate reaction principle is presented in the Supplementary Information Figs. S1.2 and S1.3.

Tests were conducted for OR and AND gates under all possible input combinations, producing the expected correct outputs in all cases (Fig. 1B), demonstrating the high accuracy of logical operations in triplex structure-based DNA logic circuits. Furthermore, the reaction half-life of each single gate was less than two minutes, showcasing the high reaction rate of this system and further validating its superior performance.

### The construction of secondary cascade utilizing the triplex structure-based DNA logic circuits

**Fig. 2:**
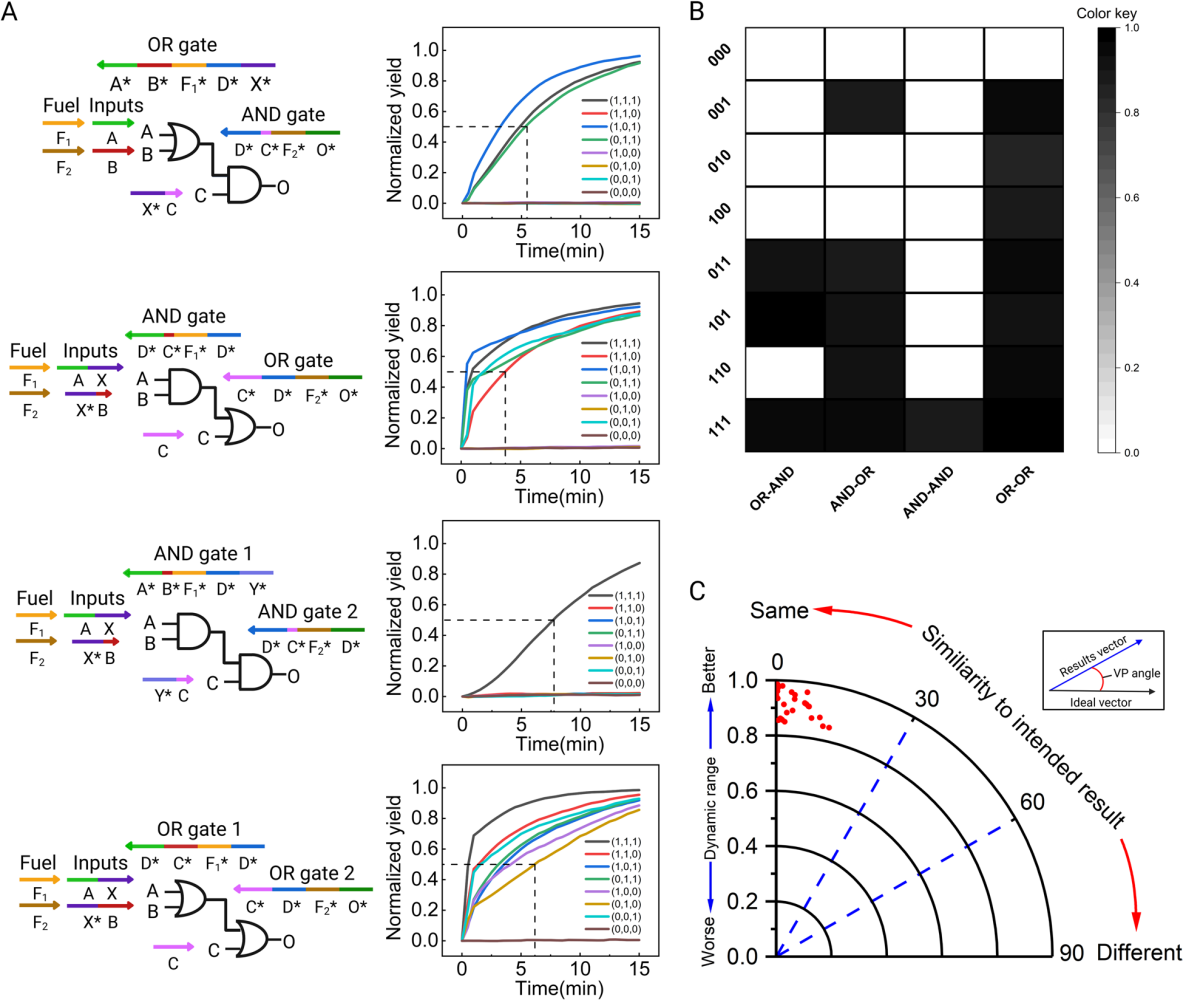
Construction of secondary cascade and their performance. **A** Composition of four secondary cascading structures (OR-AND, AND-OR, AND-AND, OR-OR) and their experimental results. **B** Summary of experimental results for all four secondary cascading structures (total of 32 results). Heat map with error bars for Fig. 2B is provided in Supplementary Information Fig. S3A. **C** Detection results of dynamic range and vector proximity for all output results, including plotting the relationship between the vector proximity (VP) angle and the corresponding dynamic range

Based on the successful construction of high-performance fundamental logic gates, we designed multiple secondary cascading circuits to demonstrate the scalability of our system. Various secondary cascading circuits (Fig. 2A) were designed according to the aforementioned principles, including OR-AND, AND-OR, AND-AND, and OR-OR configurations. All input combinations were systematically tested and produced the expected outputs (Fig. 2A). Furthermore, the fluorescence intensity measurements indicated no significant signal leakage and the longest half-completion time of less than 8 min, reflecting an extremely fast reaction rate of our system. A heatmap illustrating the results of all 32 input combinations for the four secondary cascading circuits was generated (Fig. 2B), showing close agreement with the ideal results, which highlights the high accuracy of the system's

logical computations. Additionally, dynamic range and vector proximity analyses were performed on all results (Fig. 2C), further confirming the system's computational accuracy and robustness. These results demonstrate that the system maintains high performance while retaining excellent scalability, laying a solid foundation for the development of more complex DNA circuit systems.

### The construction of more complex cascades utilizing the triplex structure-based DNA logic circuits

**Fig. 3:**
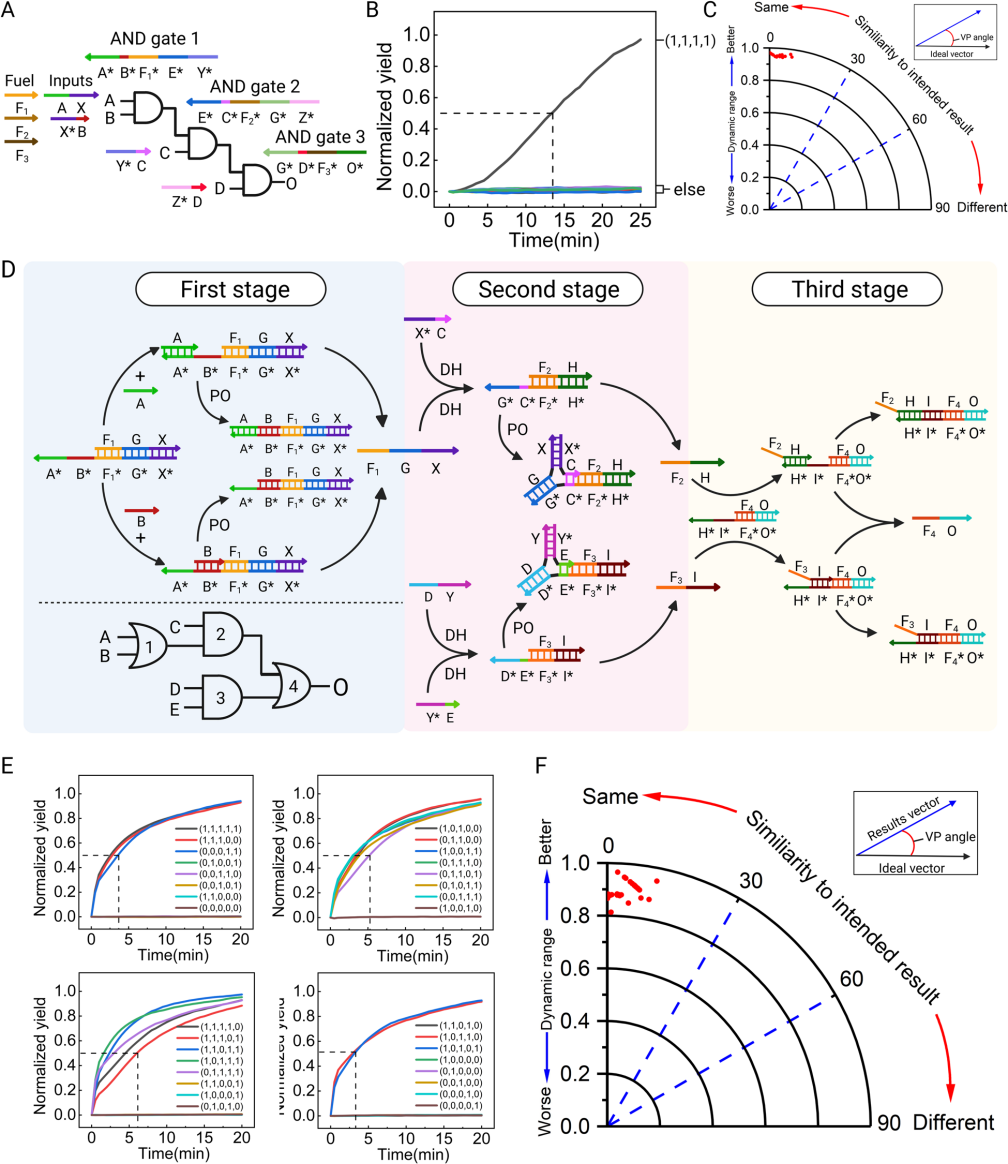
Construction and performance of tertiary cascades and the relatively complex four-gate logic circuit. **A** Schematic diagram of the three-level AND cascade design. **B** Fluorescence curves of all 16 input results for the three-level AND cascade. **C** Detection results of the dynamic range and vector proximity (VP) angle for the 16 inputs in the three-level AND cascade, with a plot showing the relationship between the VP angle and the corresponding dynamic range. **D** Schematic diagram of the relatively complex four-gate logic circuit design and the reaction process. **E** Fluorescence curves of all 32 input results for the relatively complex four-gate logic circuit. **F** Detection results of the dynamic range and vector proximity (VP) angle for all inputs in the relatively complex four-gate logic circuit

We constructed a tertiary cascade consisting of three interconnected AND gates following the successful construction of secondary cascading circuits with good computational performance (Fig. 3A). All input combinations were tested and produced the correct outputs (Fig. 3B). Fluorescence curves revealed minimal signal leakage, with clear distinctions between theoretical fluorescence-producing groups and theoretical non-fluorescence-producing groups. Dynamic range and vector proximity analyses were performed for all inputs, yielding satisfactory results (Fig. 3C). These findings suggest that our system (the triplex structure-based DNA logic circuits) can theoretically achieve higher-level AND cascades, demonstrating superior scalability and promising long-term prospects.

In this work, we designed a medium-scale four-gate circuit based on secondary and tertiary cascading to validate the high programmability of this system. The entire reaction process of relatively complex four-gate logic circuit is systematically demonstrated in Fig. 3D. The output pathway of the four-gate circuit is divided into two branches. Pathway 1: Gate 1-Gate 2-Gate 4; Pathway 2: Gate 3-Gate 4. Both pathways require an initial preparation phase to form intermediate complexes. In Pathway 1, upon the introduction of input A or input B, they bind to the corresponding domains on the intermediate complex formed by Gate 1 (A-A*, B-B*). Under the action of DNA polymerase, the strand F_1_GX is extended and dissociated. Strand F_1_GX then serves as the input for Gate 2 (AND gate), binding to input C and the intermediate complex formed by Gate 2. After extension and displacement, strand F_2_H is generated. Strand F_2_H acts as the input for Gate 4, binding to the intermediate complex formed by Gate 4 (H–H*). Further enzymatic extension and dissociation produce output O, which is visualized as fluorescent signals in the reporting stage. In Pathway 2, only when both inputs D and E are present do they bind to the intermediate complex formed by Gate 3. Enzymatic extension and dissociation generate strand F_3_I, which also serves as the input for Gate 4, binding to the intermediate complex formed by Gate 4 (I-I*). Further enzymatic extension and dissociation produce output O, which is visualized as fluorescent signals in the reporting stage.

All 32 possible inputs to the circuit were tested, yielding correct computational results with negligible leakage in the reaction system (Fig. 3E). Dynamic range and vector proximity analyses were conducted for all results (Fig. 3F), confirming the accuracy and stability of the triplex structure-based DNA logic circuit's operations. The longest half-completion reaction time for the entire circuit system was less than 7 min, demonstrating the high efficiency of the system. Compared to most current enzyme-driven DNA logic circuit systems, we have improved reaction efficiency while maintaining almost negligible leakage in the system, validating the exceptional performance and promising development potential of our system.

### Assembling square root circuit with the triplex structure-based DNA logic circuits

**Fig. 4:**
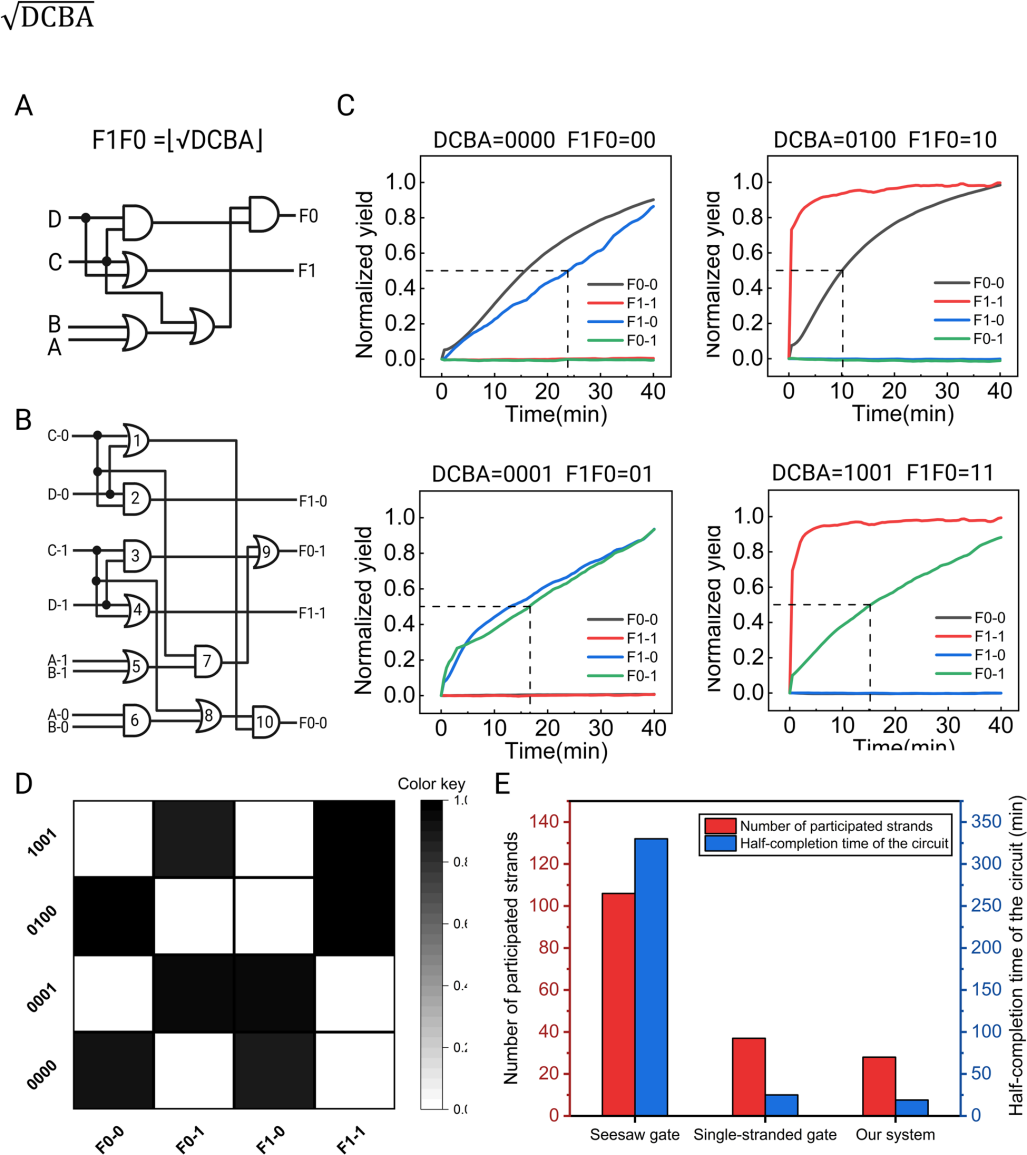
Construction of the square root circuit and their performance. **A** A square root circuit for calculating F1F0 = $\sqrt{\text{DCBA}}$. **B** A four-digit square root logic circuit based on dual-rail logic conversion. **C** Experimental results for four representative input combinations. Further results are given in Supplementary Information Fig. S6. **D** Summary of results for four representative inputs. Heat map with error bars for **D** is provided in Supplementary Information Fig. S3B. **E** Comparison of the number of square root circuit strands and half completion times (min) between our system and other mainstream systems circuit achieved a 24.3% reduction in reaction strand numbers and a 25% decrease in half-completion time in the application of square root circuits. The high-speed and low-leakage operational performance strongly validates the effectiveness of our DNA logic circuit design philosophy, which focuses on reducing strand complexity. Furthermore, it underscores the theoretical capability of minimalist DNA logic circuits to implement more complex digital algorithms, offering promising future prospects

To demonstrate the scalability of our system, we implemented a circuit consisting of ten gates to compute the square-root function of four-bit numbers where F1F0 = $\sqrt{\text{DCBA}}$. Since the single-rail system lacks a NOT gate, the original circuit was converted into a dual-rail system (Fig. 4B), where each variable in the original circuit is represented by two variables in the conversion circuit. To comprehensively evaluate the DNA logic operation performance of the square root circuit, we verified its computing performance under all 16 possible input combinations. The circuit correctly processed all cases, and we present four meaningful input combinations (0000, 0001, 0100, 1001) in Fig. 4C, while the remaining 12 inputs will be presented in Supplementary Information Fig. S6. The final data were statistically analyzed and presented as a heatmap (Fig. 4D), which closely matched the expected outcomes, confirming the system's computational accuracy. Furthermore, calculation results for all 16 input combinations reveal that minimal leakage occurred throughout the reaction system, with the longest half-completion time strictly controlled within 25 min, highlighting the outstanding performance and broad applicability potential of this system. Additionally, we compared our system with systems utilizing seesaw gate and single-stranded gate in terms of the number of DNA strands required for a four-bit square root circuit and the system's half-completion time (Fig. 4E). As shown in Fig. 4E, compared to single-stranded gate, which require fewer reaction strands and shorter reaction times, our minimalist DNA logic circuit achieved a 24.3% reduction in reaction strand numbers and a 25% decrease in half-completion time in the application of square root circuits. The high-speed and low-leakage operational performance strongly validates the effectiveness of our DNA logic circuit design philosophy, which focuses on reducing strand complexity. Furthermore, it underscores the theoretical capability of minimalist DNA logic circuits to implement more complex digital algorithms, offering promising future prospects.

## Discussion

In this article, we utilized enzyme-driven circuits as the foundation to achieve logic operations through simple concatenation of input strands with the gate strand (forming triplex structures), significantly reducing the complexity of strands (reduced by 24.3% in large-scale DNA circuit) while maintaining high reaction rates and minimal leakage, thereby enhancing the scalability of the system. Our design demonstrates ultrafast reaction kinetics while maintaining minimal leakage, as validated through single gates, multi-level cascades (secondary and tertiary), a relatively complex logic circuit, and a ten-gate square root circuit (half-completion time < 25 min). However, the stability of strand binding within the resulting triplex structure was insufficient, leading to inevitable signal loss in the system. By optimizing the input concentration, the system can still be maintained within an optimal range (Details are displayed in Supplementary Information Fig. S2.3). While ensuring the system retained extremely high reaction rates and minimal reaction leakage, we further reduced the complexity of strand requirements, showcasing exceptional scalability. Through modular design, multiple logic gates can be efficiently interconnected and operate in concert, offering a novel direction for constructing more intricate DNA computing networks. Additionally, the reduction in strand complexity directly decreased the quantity of DNA strands required, significantly lowering associated costs and diminishing the risk of strand crosstalk. This advantage is particularly critical in the construction of large-scale DNA circuits, such as applications in DNA data storage, molecular encoding, and large-scale biosensor arrays [31, 32].

Our study presents a novel enzyme-driven DNA logic circuit design capable of robust operation at both 50 °C and physiological temperature (37 °C). As experimentally validated in Supplementary Information Figs. S2.1 and S2.2, although lower temperatures lead to a reduction in reaction rate, the sequence design and logic operation remain fully compatible, while the system maintains complete logical integrity and operational reliability. This successful demonstration of temperature adaptability paves the way for the system's use in clinical and point-of-care diagnostics [6], where biocompatibility and computational accuracy are crucial.

## Supplementary Information

Additional file 1.

## Data Availability

No datasets were generated or analysed during the current study.
