# Supplementary material for: Enzyme-driven triplex structure-based DNA logic circuits
Source: J Nanobiotechnology. 2025 Dec 24;23:789. doi: 10.1186/s12951-025-03853-6 (PMC12729061; doi:10.1186/s12951-025-03853-6)
Supplement: Supplementary file 1 — Additional file 1. [file 12951_2025_3853_MOESM1_ESM.docx]

Supplementary Information for
Enzyme-driven triplex structure-based DNA logic circuits

Authors: Xiao Liu2,4,#, Jing Zhang2,5,#, Xuehao Zhang7,#,Zhuo Chen2, Shize Zhang2, Xinyu Wang6, Jiaying Liu2,5, Zhiheng Wang2, Liyi Xie2, Zhihao Ming1, Longjie Li7, Kaixiong Tao4, Xianjin Xiao2,*, Xiaoyu Zhou3,*, Yu Xie1,*
AUTHOR ADDRESS ：
1Hunan Cancer Hospital /The Affiliated Cancer Hospital of Xiangya School of Medicine, Central South University, Changsha 410000, China
2Institute of Reproductive Health, Tongji Medical College, Huazhong University of Science and Technology, Wuhan 430030, China
3Department of Biomedical Sciences and Tung Biomedical Sciences Centre, Department of Precision Diagnostic and Therapeutic Technology (CityUHKMSRI), Key Laboratory of Biochip Technology and Biotech and Health Centre (CityUSRI), City University of Hong Kong, Hong Kong 999077, China
4Department of Gastrointestinal Surgery, Union Hospital, Tongji Medical College, Huazhong University of Science and Technology, Wuhan 430030, China
5School of Pharmacy, Tongji Medical College, Huazhong University of Science and Technology, Wuhan 430030, China
6Department of Breast Surgery, Second Hospital of Jilin University, Changchun 130000, China
7School of Life Science and Technology, Wuhan Polytechnic University, Wuhan 430023, China
*Yu Xie. Email: xieyu420@sina.com
*Xianjin Xiao. Email: xiaoxianjin@hust.edu.cn
* Xiaoyu Zhou. Email: xzhou74@cityu.edu.hk
Xiao Liu# , Jing Zhang# and Xuehao Zhang#  contributed equally to this work.


S1. Molecular Design

Fig. S1.1| Molecular design of enzyme-driven DNA logic circuits. For the OR gate, all domains (A, B on the input strands, A*, B*, F*,O* on the gate strand, and F on the fuel strand) are designed to be 20 nt. To prevent spurious polymerization in larger circuits, a 5-nt poly-A tail is appended to the 3' end of both the gate and quenching strands. For the AND gate, domains B and B* are set to 6 nt, while domains X and X* retain a length of 20 nt. It is worth noting that A single adenine (A) spacer is inserted at key junctions: between domains A and X on the input A strand, between X* and B on the input B strand, and between B* and A* on the gate strand, to alleviate steric hindrance.


Fig. S1.2| Molecular mechanism of the OR gate, AND gate, and reporter module. Schematic illustration of the operational principles for the OR gate, AND gate, and reporter module under specific input conditions. The specific mechanisms of DNA hybridization (DH) and polymerization (PO) reactions are elaborated. The reaction details of the OR gate for the input condition A=1, B=1 are illustrated in Figure S1.3.


Fig. S1.3| Reaction Mechanism of the OR Gate with 11 Inputs. Supplementary illustration focusing on the OR gate when both inputs A and B are present (A=1, B=1). The binding order of inputs determines the reaction pathway. Priority binding and extension of A-A* produces output FO stoichiometrically, whereas priority binding and extension of B-B* yields an equivalent amount of FO plus an intermediate (BFO-O*F*B*A*) that is capable of responding to the A input to generate output BFO. The pre-extension process of the F strand and the DNA hybridization (DH) process are omitted from this diagram for clarity.


S2. Optimize experiments
As shown by the results in Figure S2.1, leakage signals were observed in the B-only experimental groups with 7-nt and 8-nt B domains at 50°C. According to our analysis, this leakage may originate from the following mechanism: although the B–B* interaction at this length setting does not form a stable duplex, random collisions between DNA strands in solution can lead to transient B–B* binding between input strand B and the gate strand. This transient binding, combined with the high catalytic activity of Bst DNA polymerase, creates conditions conducive to strand extension, thereby inducing leakage. Moreover, the length of the B domain in input strand B is closely related to the probability of such transient binding occurring.

At 37°C, the lower temperature improves DNA hybridization stability but reduces the catalytic activity of the strand-displacing polymerase. According to the leakage mechanism initiated by random collisions and enzymatic catalysis, although the probability of transient B-B* binding increases for the same B domain length, the decreased enzyme activity lowers the probability that such binding leads to leakage. Therefore, after the temperature decrease, the minimal B domain length required to observe clear leakage will reflects a combined effect of these factors.

The comparison of the experimental results at 50°C and 37°C further supports this observation. In the case where both A and B inputs are present (Figure S2.1A and Figure S2.2A), the reaction rate at 37°C is noticeably slower than that at 50°C, while the signal plateau is higher. In setups with only B input (Figure S2.1B and Figure S2.2B), observable leakage occurs only when the B domain reaches 7 nt, under both temperature conditions.

Taken together, these findings confirm that a B domain length of 6 nt remains viable at physiological temperature. Thus, our circuit design and sequences can be directly applied under 37°C condition. Although the reaction rate is somewhat lower compared to that at 50°C, the operational performance and logical correctness of the system remain fully intact. These results strongly validate the potential of our design for use in biological environments.


Fig. S2.1| Optimization of B domain length for AND gates at 50°C. The effect of eight different B domain lengths on the signal (both loss and leakage) in the AND gate was tested. (A) Inputs A and B were added to the system simultaneously, and the fluorescence loss was shown by the fluorescence signal. Within a certain range, when the length of the B domain is longer, the fluorescence signal is higher and the corresponding signal loss is lower. (B) Addition of only input B to the system and display of signal loss by fluorescence signal. Those with smaller fluorescence loss lengths (4-8 nt) were selected for the experiment, among which 7 nt and 8 nt showed obvious signal leakage, and finally 6 nt was selected for the following experiments.


Fig. S2.2| Optimization of B domain length for AND gates at 37°C. The effect of different B domain lengths on signal performance (loss and leakage) in the AND gate was evaluated at 37°C. (A) Fluorescence signal loss when both inputs A and B are added simultaneously, tested across various B domain lengths(1-7nt). (B) Signal leakage when only input B is added, tested under the same conditions. The results guide the selection of an optimal B domain length to minimize undesired signal effects in physiological temperatures.


Fig. S2.3| AND gate input B concentration optimization. To minimize system signal loss, the input B concentration was increased and experiments were performed on a range of concentrations. (A) While keeping the concentration of Input A constant at 1×, we gradually increased the concentration of Input B from 1× to 1.5×. The fluorescence signal of the system showed a significant increase as the concentration of Input B rose from 1× to 1.3×. However, beyond 1.3×, the fluorescence signal remained relatively stable despite further increases in Input B concentration. (B) When only Input B was added, its concentration ranges from 1× to 1.5× did not result in any noticeable signal leakage. Considering the relatively simpler composition of the single-gate system and to prevent potential crosstalk-induced leakage in larger circuits, we ultimately selected Input B concentration at 1.3×. Measurement results indicated a signal loss rate of approximately 17% within the single gate.


S3. Statistical results including error range

Fig. S3| Statistical results with error ranges for the two-level cascade and square root circuits. Heat maps displaying the fluorescence outputs of the logic circuits. Values are expressed as the mean±standard deviation (SD) from replicate experiments. (A) Results for the two-level cascade circuit, showing the output signals under different input combinations. (B) Results for the square root circuit, illustrating the computed square root values based on input patterns. The error ranges provide insight into the reproducibility and variability of the experimental data.


S4. Logic circuit configuration

Fig.S4| Logical structure diagram and corresponding serial numbers of each gate.


S5. Truth tables for all logic circuits 

Fig.S5.1| Truth tables for single gates and multi-level cascades utilized in the experiment.


Fig.S5.2| Truth tables for the relatively complex four-gate logic circuit and the square root circuit utilized in the experiment.

S6. Fluorescence experiment results of the square-root circuit 

Fig.S6.1| Fluorescence experiment results of the square-root circuit (part 1).


Fig.S6.2| Fluorescence experiment results of the square-root circuit (part 2).


Fig.S6.3| Fluorescence experiment results of the square-root circuit (part 3).


S7. DNA sequences Information
Table S1.1 Sequences of the OR gate
Number	Strand	Sequence
1	Rep-FAM	GACTGGTTAGATGGATGTGATGTGA-(FAM)
2	Rep-BHQ	(BHQ1)-TCACATCACATCCATCTAACCAGTCAACACACTCAATCCTCACTCAATACCTATCA
3	G	CTCAATCCTCACTCAATACCTATCACTCTACTACTACATAACCTATCAAGCTACTGAACTATTCTCATACTCACTAACAACTAGATCTGTACTAC
4	F	TAGGTTATGTAGTAGTAGAG
5	A	GTAGTACAGATCTAGTTGTTAGTGA
6	B	GTATGAGAATAGTTCAGTAGCTTGA

 
Table S1.2 Sequences of the AND gate
Number	Strand	Sequence
1	Rep-FAM	GACTGGTTAGATGGATGTGATGTGA-(FAM)
2	Rep-BHQ	(BHQ1)-TCACATCACATCCATCTAACCAGTCAACACACTCAATCCTCACTCAATACCTATCA
3	G	CTCAATCCTCACTCAATACCTATCACTCTACTACTACATAACCTACTACTCAACATCAAGTACGTATACAATTCAACT
4	F	AGTAGTAGGTTATGTAGTAGTAGAG
5	A	AGTTGAATTGTATACGTACTAATCAGAATCGAGCTAAGTAG
6	B	CTACTTAGCTCGATTCTGATAGATGTT

 
Table S2.1 Sequences of the OR-AND cascade
Number	Strand	Sequence
1	Rep-FAM	GACTGGTTAGATGGATGTGATGTGA-(FAM)
2	Rep-BHQ	(BHQ1)-TCACATCACATCCATCTAACCAGTCAACACACTCAATCCTCACTCAATACCTATCA
3	G1	CTACTTAGCTCGATTCTGATTAGTACGTATACAATTCAACTAAAGACTTCACTTAGGTAACAGACTGCTATACTCTCAAACACCAAGTCCTAGAACAGATC-AAAAA
4	F1	GTTACCTAAGTGAAGTCTTT
5	A	GATCTGTTCTAGGACTTGGT
6	B	GTTTGAGAGTATAGCAGTCT
7	G2	CTCAATCCTCACTCAATACCTATCACTCTACTACTACATAACCTACTACTCAACATCAAGTACGTATACAATTCAACT-AAAAA
8	F2	AGTAGTAGGTTATGTAGTAGTAGAG
9	C	CTACTTAGCTCGATTCTGATAGATGTT

 
Table S2.2 Sequences of the AND-OR cascade
Number	Strand	Sequence
1	Rep-FAM	GACTGGTTAGATGGATGTGATGTGA-(FAM)
2	Rep-BHQ	(BHQ1)-TCACATCACATCCATCTAACCAGTCAACACACTCAATCCTCACTCAATACCTATCA
3	G1	TCAAGCTACTGAACTATTCTCATACAGATACATACTAGCATCAGACAACATCAAGTACGTATACAATTGAACT
4	F1	TCTGATGCTAGTATGTATCT
5	A	AGTTCAATTGTATACGTACTAATCAGAATCGAGCTATCTAG
6	B	CTAGATAGCTCGATTCTGATAGATGTTG
7	G2	CTCAATCCTCACTCAATACCTATCACTCTACTACTACATAACCTATCAAGCTACTGAACTATTCTCATACTCACTAACAACTAGATCTGTACTAC
8	F2	TAGGTTATGTAGTAGTAGAG
9	C	GTAGTACAGATCTAGTTGTTAGTGA


Table S2.3 Sequences of the AND-AND cascade
Number	Strand	Sequence
1	Rep-FAM	GACTGGTTAGATGGATGTGATGTGA-(FAM)
2	Rep-BHQ	(BHQ1)-TCACATCACATCCATCTAACCAGTCAACACACTCAATCCTCACTCAATACCTATCA
3	G1	CTAGTTACCACAGTACAGATTAGTACGTATACAATTCAACTGATAGCTACATGATGACAGATACACCTAGTCTAGTCTTCGTATCAAGT
4	F1	TCTGTCATCATGTAGCTATC
5	A	ACTTGATACGAAGACTAGACACTTGATCAGACTTGTATCAC
6	B	GTGATACAAGTCTGATCAAGAAGGTGTA
7	G2	CTCAATCCTCACTCAATACCTATCACTCTACTACTACATAACCTACTACTCAACATCAAGTACGTATACAATTCAACT
8	F2	AGTAGTAGGTTATGTAGTAGTAGAG
9	C	CTAGTTACCACAGTACAGATAGATGTTG

 
Table S2.4 Sequences of the AND-AND-AND cascade
Number	Strand	Sequence
1	Rep-FAM	GACTGGTTAGATGGATGTGATGTGA-(FAM)
2	Rep-BHQ	(BHQ1)-TCACATCACATCCATCTAACCAGTCAACACACTCAATCCTCACTCAATACCTATCA
3	G1	GTGATACAAGTCTGATCAAGTGTCTAGTCTTCGTATCAAGTTCTCTCATACATGACTCAGATCTGTAGAACACTATCGATGTAATCTCT
4	F1	TCTGAGTCATGTATGAGAGA
5	A	AGAGATTACATCGATAGTGTAACAAGTTAGCTGACTGAACA
6	B	TGTTCAGTCAGCTAACTTGTACTACAGA
7	G2	CTAGTTACCACAGTACAGATTAGTACGTATACAATTCAACTGATAGCTACATGATGACAGATACACCTAGTCTAGTCTTCGTATCAAGT
8	F2	TCTGTCATCATGTAGCTATC
9	C	GTGATACAAGTCTGATCAAGAAGGTGTA
10	G3	CTCAATCCTCACTCAATACCTATCACTCTACTACTACATAACCTACTACTCAACATCAAGTACGTATACAATTCAACT
11	F3	AGTAGTAGGTTATGTAGTAGTAGAG
12	D	CTAGTTACCACAGTACAGATAGATGTTG

 
Table S3.1 Sequences of a relatively complex circuit of four gates
Number	Strand	Sequence
1	Rep-FAM	GACTGGTTAGATGGATGTGATGTGA-(FAM)
2	Rep-BHQ	(BHQ1)-TCACATCACATCCATCTAACCAGTCAACACACTCAATCCTCACTCAATACCTATCA
3	G1	AGTCTAGTCTACAGGTCTCATAATGTGGATCGTCTGTCTCATGGTACTCTATGATGATCGAAGTCAGCTATACTCTCAAACACCAAGTCCATGAACACTAC
4	F1	TCGATCATCATAGAGTACCA
5	A	GTAGTGTTCATGGACTTGGT
6	B	GTTTGAGAGTATAGCTGACT
7	G2	TCACTAACAACTAGATCTGTACTACACTGTTCTCGTATGACTACAACAACTGAAATGTGGATCGTCTGTCTCA
8	F2	TGTAGTCATACGAGAACAGT
9	C	AGTCTAGTCTACAGGTCTCAACAGTTG
10	G3	TCAAGCTACTGAACTATTCTCATACAGATACATACTAGCATCAGACAACATCAAGTACGTATACAATTGAACT
11	F3	TCTGATGCTAGTATGTATCT
12	D	AGTTCAATTGTATACGTACTAATCAGAATCGAGCTATCTAG
13	E	CTAGATAGCTCGATTCTGATAGATGTT
14	G4	CTCAATCCTCACTCAATACCTATCACTCTACTACTACATAACCTATCAAGCTACTGAACTATTCTCATACTCACTAACAACTAGATCTGTACTAC
15	F4	TAGGTTATGTAGTAGTAGAG

 
Table S4.1 Sequences of the square-root circuit
Number	Strand	Sequence
1	RepF1-0	GACTGGTTAGATGGATGTGATGTGA-(FAM)
2	RepQ1-0	(BHQ1)-TCACATCACATCCATCTAACCAGTCAACACACTCAATCCTCACTCAATACCTATCA
3	RepF0-1	GTATGTAGAGAGATAAGTTG-(ROX)
4	RepQ0-1	(BHQ2)-CAACTTATCTCTCTACATAC-AAA-CTACGATTCTCACCTTTCCACTACAATCCT-AAAAA
5	RepF1-1	GAGTTGTCTGAAGGTTGATG-(HEX)
6	RepQ1-1	(BHQ1)-CATCAACCTTCAGACAACTC-AAA-CAATCCTTCTACGATCCTACTACCTTACAC-AAAAA
7	RepF0-0	TAGCTTAGAGTACGATGATG-(Cy5)
8	RepQ0-0	(BHQ3)-CATCATCGTACTCTAAGCTA-AAA-ACTACCTACACTATCTACTAACTCACTGAC-AAAAA
9	G1	GTACTATGCTCATGTCTGATTAGTCACTATACAATTCAACTCTCTACTTCTACATGTACAAACCACTTCTTGAACTGACTGCATCTTCTTCATGTGATACA-AAAAA
10	F1	TTGTACATGTAGAAGTAGAG
11	G2	ATTCTCACCTTTCCACTACAATCCTGATAGCTACATGATGACAGATACCACTAGTCTACTCTTCGTATCAAGT-AAAAA
12	F2	TCTGTCATCATGTAGCTATC
13	G3	AACAACTAGAACGTTATCCATATGCACTGATCATAACCTACACTCTAACATTGAGATCTAGACACGAT-AAAAA
14	F3	TAGGTTATGATCAGTGCATA
15	G4	CTTCTACGATCCTACTACCTTACACACACATCTCAGTAGACTACAACATACGATCAATAGAACAGACTCTATGTATCTTGTCGAC
16	F4	TGTAGTCTACTGAGATGTGT
17	G5	ATCATCTCTTGAACTGACTTGTATCACAAAGACTTCACTTAGGTAACAGACTGCTATACTCTCAAACACCAAGTCCTAGAACACTAC-AAAAA
18	F5	GTTACCTAAGTGAAGTCTTT
19	G6	AGTTGACTAGACTACTGTACTCCATCATACTTGACTCAGATCTGTAGAACTCTTTCGATGTAATCTCT-AAAAA
20	F6	TCTGAGTCAAGTATGATGGA
21	G7	TGACCGAACTAATCTCTTACTGTGTTGCTATGTTAACGTGGATCATCAGTCTACTCTTCGTATCAAGT-AAAAA
22	F7	CACGTTAACATAGCAACACA
23	G8	AACATCTATCAGACATGAGCATAGTACTGGATTGCATTCTTGTTCGAAGTTGACTAGACTACTGTACACATACGATCAATAGAACAG-AAAAA
24	F8	TCGAACAAGAATGCAATCCA
25	G9	CTATCTACTAACTCACTGACAGTCTAAACATCCTTCTATCAACAACTAGAACGTTATCCATGACCGAACTAATCTCTTAC-AAAAA
26	F9	GATAGAAGGATGTTTAGACT
27	G10	CTCAATCCTCACTCAATACCTATCACTGTACTACTACTACTACTACTACTCAACATCAAGTCACTATACAATTCAACT-AAAAA
28	F10	AGTAGTAGTAGTAGTAGTAGTACAG
29	C-0	ACTTGATACGAAGAGTAGACACTTGAACTGACTTGTATCACATGAAGAAGATG
30	D-0	GTGATACAAGTCAGTTCAAGAAGTGGT
31	C-1	ATCGTGTCTAGATCTCAATGAGTATCTTGTCGACTGTTCTATTGATCGTATGT
32	D-1	TAGAACAGTCGACAAGATACATAGAGT
33	A-1	GTAGTGTTCTAGGACTTGGT
34	B-1	GTTTGAGAGTATAGCAGTCT
35	A-0	TGTTCAGTCAGCTAACTTGTACTACAG
36	B-0	AGAGATTACATCGAAAGAGTAACAAGTTAGCTGACTGAACA-AAAAA


Table S5.1 Sequences of the test experiment
1	Rep-FAM	TGATAGGTATTGAGTGAGGATTGAG-(FAM)
2	Rep-BHQ	(BHQ1)-CTCAATCCTCACTCAATACCTATCACTCTACTACTACATAACCTACTACTCAACATCAAGTACGTATACAATTCAACT
3	I1	AGTTGAATTGTATACGTACTAATCAGAATCGAGCTAAGTAG
4	I2-8nt	CTACTTAGCTCGATTCTGATAGATGTTGA
5	I2-7nt	CTACTTAGCTCGATTCTGATAGATGTTG
6	I2-6nt	CTACTTAGCTCGATTCTGATAGATGTT
7	I2-5nt	CTACTTAGCTCGATTCTGATAGATGT
8	I2-4nt	CTACTTAGCTCGATTCTGATAGATG
9	I2-3nt	CTACTTAGCTCGATTCTGATAGAT
10	I2-2nt	CTACTTAGCTCGATTCTGATAGA
11	I2-1nt	CTACTTAGCTCGATTCTGATAG
12	I2-neg	CTACTTAGCTCGATTCTGATA-AAAAA
